# Development and characterization of novel microsatellite loci in the native tree frog species, *Polypedates braueri* and cross-species amplification in the alien species, *P. megacephalus*, in Taiwan

**DOI:** 10.3897/BDJ.13.e160332

**Published:** 2025-09-04

**Authors:** Yuan-Cheng Cheng, Yi-Ju Yang, Yi-Huey Chen

**Affiliations:** 1 Department of Life Science, National Taiwan Normal University, Taipei, Taiwan Department of Life Science, National Taiwan Normal University Taipei Taiwan; 2 Department of Natural Resources and Environmental Studies, National Dong-Hwa University, Hualien, Taiwan Department of Natural Resources and Environmental Studies, National Dong-Hwa University Hualien Taiwan; 3 Department of Life Science, Chinese Culture University, Taipei, Taiwan Department of Life Science, Chinese Culture University Taipei Taiwan

**Keywords:** amphibian, anuran, microsatellite, Rhacophoridae, SSR marker

## Abstract

*Polypedates megacephalus* is an alien species first recorded in 2006 in Taiwan. The expanding population of *P. megacephalus* poses potential threats to the native frog species, especially to the closely-related species *P. braueri*. To detect genetic diversity and analyse population structures of both native and alien *Polypedates* species in Taiwan, this study aimed to isolate microsatellite markers in *P. braueri* and test their cross-species amplification in the alien species *P. megacephalus*. We successfully amplified and characterised 10 polymorphic microsatellite loci in *P. braueri*. The number of alleles per locus ranged from 2 to 19 and no locus showed evidence of a null allele. The observed and expected heterozygosity ranged from 0.200 to 1.000 and 0.180 to 0.916, respectively and each locus was detected in Hardy-Weinberg equilibrium. Cross-amplification in *P. megacephalus* was successfully performed in seven out of 10 loci. Amongst these seven loci, five exhibited polymorphism and two showed no variation. These microsatellite markers will be helpful for further population genetics research on *Polypedates* species in Taiwan.

## Introduction

The number of documented invasive alien amphibian species has been steadily increasing worldwide, a trend strongly associated with the growing frequency of cargo transport, horticultural trade, the pet trade and other human activities (Kraus 2009a, Kraus 2009b, Fonseca et al. 2019, Mohanty and Measey 2019, González-Sánchez et al. 2021). Before 1850, records of such invasions were scarce (Kraus 2009a). According to the 2016 IUCN statistics on global invasive alien species, out of 419 alien animal species, 13 invasive amphibian species were documented with available studies (Kraus 2015, Smith 2020). In recent years, however, additional species not previously included in the lists have been recognised as invasive — for example, the black-spined toad (*Duttaphrynus melanostictus*) (Marshall et al. 2018, Licata et al. 2019, Licata et al. 2022). Some species remain unrecognised as harmful invasive species due to limited evidence or lack of impact assessment (Measey et al. 2016, Kumschick et al. 2017, Kumschick et al. 2024); as research continues, more species are likely to be identified as invasive aliens.

Islands exhibit significantly higher invasibility — the capacity of alien species to colonise and establish populations in non-native geographic ranges — compared to mainland regions (D'Antonio and Dudley 1995, Capinha et al. 2017). Likewise, island ecosystems are substantially more vulnerable to biological invasions, meaning they face more significant risks of invasive alien species causing population declines, threatening native biodiversity or even driving native species to extinction (D'Antonio and Dudley 1995, Sax et al. 2002, Sax and Gaines 2008, Russell and Kueffer 2019, Dueñas et al. 2021). In recent decades, there has been a notable increase in reports documenting the impacts of invasive alien amphibians on island ecosystems (Dueñas et al. 2021). Historical examples include the introduction of the Puerto Rican coqui frog (*Eleutherodactylus coqui*) in Hawaii (Beard and Pitt 2005, Beard et al. 2009, Choi and Beard 2012), the greenhouse frog (*E. planirostris*) in Hawaii and Guam (Olson et al. 2012), the black-spined toad (*Duttaphrynus melanostictus*) in Madagascar (Marshall et al. 2018, Licata et al. 2019, Licata et al. 2022) and the cane toad (*Rhinella marina*) in the Philippines (Harvey et al. 2021).

**Figure 1.**
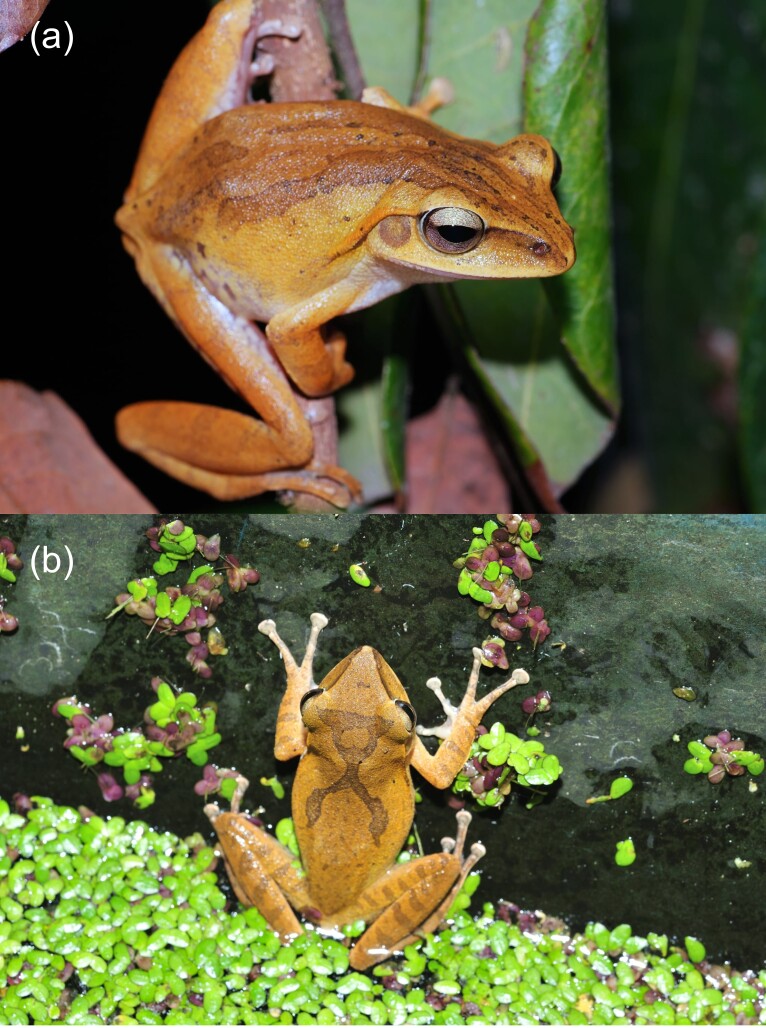
The native tree frog species, *Polypedates braueri* (**a**) and the congeneric alien species, *P. megacephalus* (**b**), in Taiwan (photo by Peng-Hsiang Lee).

*Polypedates megacephalus* (Anura, Rhacophoridae), an invasive alien species in the main island of Taiwan (Lee et al. 2019), was first recorded in Taichung, a city in central Taiwan, in 2006 (Yang and Gong 2014, Lee et al. 2019). Since then, the population of *P. megacephalus* has expanded quickly and spread across lowland areas in Taiwan (Yang et al. 2014, Lee et al. 2019). *P. megacephalus* poses threats to native species in Taiwan. It has been documented to prey on three sympatric native amphibians and reptiles, including *Microhyla fissipes*, *Japalura swinhonis* and *Gekko hokouensis* (Chen 2014). Although these prey items constitute a relatively small proportion of its diet, such predation may nonetheless represent a substantial threat. Additionally, *P. megacephalus* exhibits a broad dietary niche, consuming a high proportion of native invertebrates (Chen 2014), which may alter the composition of invertebrate communities. Furthermore, its diet overlaps with those of several sympatric anuran species (Chen 2014), which is presumed to lead to trophic competition. In Taiwan, *P. megacephalus* shares similar morphology, breeding phenology (breeding from April to September or October) and breeding habitats (oviposition on vegetation near still-water bodies such as ponds, reservoirs and ditches) with the congeneric native species *P. braueri* (Fig. 1) (Kuraishi et al. 2011, Yang et al. 2014). However, *P. megacephalus* demonstrates a higher reproductive potential, with an average clutch size of 639 eggs compared to 349 eggs in *P. braueri* (Wu et al. 2010) and its tadpoles exhibit a competitive advantage under food-limited conditions (Fang 2022). These traits may enable *P. megacephalus* to outcompete *P. braueri*, potentially resulting in the displacement of the native species through niche replacement.

Microsatellite loci, as high-resolution nuclear genetic markers, remain valuable for population and conservation genetics studies of wildlife (Hauser et al. 2021). To analyse and compare the genetic diversity and the population structures of both native and alien *Polypedates* species in Taiwan, this study aimed to isolate and characterise novel microsatellite markers in *P. braueri* and test their cross-species amplification in the alien species *P. megacephalus*.

## Materials and methods

**Samples and DNA extraction**

We collected tissues of *P. braueri* from 30 adult individuals in the Hua-Lin Experimental Forest of Chinese Culture University, New Taipei City, Taiwan (24°54'N, 121°34'E, 200-600 m a.s.l., 92 hectares) in 2013. We visually searched for adult frogs around the pools at the Experimental Forest with flashlights. When we found the frogs, we toe-clipped each individual (Donnelly et al. 1994, Phillott et al. 2007) and preserved the clipped segments of each frog’s toe in 95% ethanol individually for DNA extraction. We did not find any *P. megacephalus* at the Experimental Forest during 2012-2014. The toe tissues of 43 *P. megacephalus* individuals were collected separately from New Taipei City (10), Taichung City (23), Changhua County (5) and Yunlin County (5) in 2013.

Total genomic DNA was extracted from the toe tissue of each individual using the MasterPure™ Complete DNA and RNA Purification Kit (EPICENTRE^®^ Biotechnologies) according to the manufacturer’s protocols. DNA was re-suspended in 100 μl TE buffer (10 mM Tris, 1 mM EDTA, pH 8.0) and stored at -20°C.

**Searching for microsatellite loci and primer design**

One of the genomic DNA samples of *P. braueri* was used for partial library preparation and microsatellites were isolated from the total DNA using an enrichment protocol (Glenn and Schable 2005, Senan et al. 2014). The set of oligonucleotide probes used for hybridisation included (AC)_10_, (TC)_10_, (AT)_15_, (TACA)_7_, (CTAT)_7_ and (AAAG)_7_. A total of 576 positive clones were obtained, of which 220 clones with insert lengths greater than 300 bp were sequenced. Microsatellite regions were searched for amongst sequences with high-quality reads. Thirty-five primer pairs of microsatellite loci with enough flanking sequences were designed by Primer 3 (Koressaar and Remm 2007, Untergasser et al. 2012).

**PCR amplification and genotyping**

We individually tested the polymerase chain reaction (PCR) amplification conditions of the loci for an initial set of eight *P. braueri* samples. Each microsatellite locus was amplified in a 5-μl PCR mixture containing 1 μl of template DNA, 0.25 units of GoTaq^®^ Flexi DNA polymerase (Promega), 2-2.5 mM of MgCl_2_ (Promega), 0.1-0.15 mM of dNTP (Amersham, GE), 1.0 μl of 5×Colorless GoTaq^®^ Flexi Buffer (pH 8.5, Promega) and 0.1 μM each of forward and reverse primer. PCR amplification was performed in a thermal cycler (Eppendorf Mastercycler^®^ gradient). The thermal profiles were heated to 95^°^C for 5 min, followed by 35 cycles of denaturation at 95^º^C for 30 s, annealing at testing/optimal temperature for each locus for 30 s or 35 s and extension at 72^º^C for 30 s. The thermal profiles were then heated to a final extension step at 72^º^C for 10 min. The loci that were amplified successfully for eight *P. braueri* samples were subsequently used to test amplification and polymorphisms for all DNA samples of *P. braueri* and *P. megacephalus*.

All PCR amplicons were subjected to capillary electrophoresis on an ABI 3730 automated sequencer, with GeneScan™ LIZ 600 (Applied Biosystems™) employed as an internal size standard. For each locus, either the forward or reverse primer was end-labelled with one of four fluorescent dyes (FAM, NED, PET or VIC). PCR amplicons from two to three loci were pooled before electrophoresis and fluorescent labelling enabled unequivocal discrimination of loci during allele size scoring. Allele sizes were scored using the software GeneMarker^®^ ver. 2.4 (SoftGenetics, LLC.). Two authors (YCC and YHC) independently checked the scoring and if any inconsistencies were found in specific loci, the PCR, genotyping and allele scoring were repeated once. We excluded the individual samples that failed to be amplified or obtained consistent allele scores.

**Genotypic analysis**

We separately characterised the polymorphic microsatellite loci in *P. braueri* and *P. megacephalus*. The number of alleles (N_A_), effective alleles (N_e_), observed heterozygosity (H_O_) and expected heterozygosity (H_e_) of each locus were calculated using GenAlEx ver. 6.5 (Peakall and Smouse 2006, Peakall and Smouse 2012). The theoretical expected probability of identity (PI) — defined as the likelihood that two individuals share the same multilocus genotype by chance — was also calculated using GenAlEx ver. 6.5 to assess the discriminatory power of the combined polymorphic loci (Peakall and Smouse 2006, Peakall and Smouse 2012). A lower PI value indicates higher discriminatory power of the genetic markers, meaning there is a very small chance of mistakenly identifying two different individuals as genetically identical (Waits et al. 2001). Statistical tests for deviations from the Hardy-Weinberg equilibrium and linkage disequilibrium for each locus of *P. braueri* were performed using Genepop on the web ver. 4.7 (Raymond and Rousset 1995, Rousset 2008). Null alleles for these loci were checked by MICRO-CHECKER ver. 2.2.3 (Van Oosterhout et al. 2004). Detections of deviations from the Hardy-Weinberg equilibrium, linkage disequilibrium and null alleles for the loci of *P. megacephalus* were not performed because of the multiple population origins.

## Results

In *P. braueri*, a total of 10 microsatellite loci, which were successfully amplified, exhibited polymorphism (Table 1). The sequences of these microsatellite loci are available at the National Center for Biotechnology Information website (NCBI; http://www.ncbi.nlm.nih.gov) and can be found using the GenBank accession numbers provided in Table 1. The characterisation included 25 samples successfully amplified across all 10 loci. The number of alleles (N_A_) per locus varied from 2 to 19, with a mean of 9.8 and the observed (H_o_) and expected heterozygosity (H_e_) ranged from 0.200 to 1.000 and 0.180 to 0.916, respectively (Table 1). No statistically significant deviations from the Hardy-Weinberg equilibrium were detected and no locus showed evidence of a null allele. Significant linkage disequilibrium was observed between Pb327 and both Pb284 and Pb360 (P < 0.00022 for both comparisons after Bonferroni correction). If Pb327 was excluded, no linkage disequilibrium between any pair of loci was detected. No pairs of individuals were found to share identical multilocus genotypes. The probability of identity (PI) based on the combination of the 10 loci was 4.8 × 10⁻¹³ and the PI of the nine loci (excluding Pb327) was 3.0 × 10^⁻11^, indicating an extremely low likelihood that two individuals would share the same multilocus genotype.

The loci isolated from *P. braueri* were used for cross-species amplifications on 43 individual samples of *P. megacephalus*. Amplifications were successful in seven out of 10 loci (Table 1). Amongst these seven loci, five (Pb168, Pb214, Pb284, Pb293, Pb327) exhibited polymorphism and two (Pb213, Pb360) showed no variation. We analysed data from 27 *P. megacephalus* individuals that were successfully genotyped at all five polymorphic loci. The number of alleles per locus ranged from 4 to 11, with a mean of 8.0 (Table 2). Three loci (Pb284, Pb293, Pb327) exhibited fewer alleles than those observed in *P. braueri*. The observed and expected heterozygosity ranged from 0.185 to 0.741 and 0.370 to 0.848, respectively (Table 2). The probability of identity (PI) based on the combination of these five loci was 8.9 × 10^⁻5^.

## Discussion

In *P. braueri*, Pb327 showed significant linkage disequilibrium with both Pb284 and Pb360, suggesting that Pb327 may not function as an independent marker when used in combination with these loci. However, this observed linkage disequilibrium may be an artefact of the small dataset or limited population size (Slatkin 2008). In future studies, researchers could consider selecting loci that consistently exhibit independence across larger and more diverse populations to enhance the reliability of genetic analyses. The extremely low probability of identity (PI) in *P. braueri* (3.0 × 10⁻¹¹ based on nine loci, excluding Pb327 and 4.8 × 10⁻¹³ based on ten loci) indicates that the microsatellite markers developed in this study provide a high level of resolution, making them suitable for both individual-level (e.g. individual identification, parentage analysis) and population-level (e.g. conservation genetics, population structure analysis) genetic studies (Waits et al. 2001).

Amongst the seven loci that were successfully cross-amplified in *P. megacephalus*, two were monomorphic and three exhibited a reduced number of alleles compared with those in *P. braueri*. The combination of five polymorphic loci yielded a higher probability of identity (PI = 8.9 × 10⁻⁵) than that for the same set of loci analysed in *P. braueri* (PI = 5.3 × 10⁻⁷, N = 30). The comparatively lower genetic diversity — evidenced by both the reduced number of alleles and elevated PI — may reflect factors such as uneven allele frequency distribution, the presence of close relatives in the samples or a bottleneck event associated with the invasion process in *P. megacephalus* (Waits et al. 2001). Moreover, given that observed PIs are often lower than theoretical expectations in various animal studies (Waits et al. 2001), further research is needed to determine whether these five microsatellite loci provide sufficient resolution for individual-level genetic applications in *P. megacephalus* populations in Taiwan.

Overall, the polymorphic microsatellite markers developed in this study will benefit ongoing research on conservation and population genetics by enabling cross-species comparisons between native and alien *Polypedates* species in Taiwan, particularly with regard to genetic diversity, population structure and potential introgression.

## Figures and Tables

**Table 1.** Characteristics of 10 polymorphic microsatellite loci in *Polypedates braueri* (N = 25) and the results of cross-species amplification in *P. megacephalus*.

Locus Name	GenBank accession #	Primer sequences (5'-3')	Repeat motif	Ta (°C)	Allele Size range (bp)	N_A_	N_e_	Ho	He	P_HWE_	Success in cross-species amplification
Pb168	KP780884	F: tcaccaagaactttggctgtgcc	(TA)_6_(CA)_4_TA(TACA)_5_	TD 56-52	113, 131	2	1.220	0.200	0.180	1.0000	33
		R: ggtgcactcacttttgtggga									
											
Pb213	KT699110	F: taactccagcactgctctgc	(AT)_4_(CA)_13_(AT)_8_(CA)_3_	53	127,133	2	1.814	0.360	0.449	0.3770	10
		R: gcctggtttccataggtgag									
											
Pb214	KP780885	F: gcaattggcagcctcatcc	(CA)_18_	56	168-194	8	5.556	1.000	0.820	0.0022	42
		R: tctccctatgggtgtgcca									
											
Pb250	KP780886	F: ccattcctcagggctaactcg	(TG)_21_GTCG(CA)_4_	52	146-194	11	5.144	0.880	0.806	0.1339	0
		R: gggtgagggcagtcagcaa									
											
Pb284	KP780887	F: acttgatgcaaataagtcacagca	(GATA)_20_	57	235-319	15	11.905	0.920	0.916	0.0241	38
		R: acatcgaaatggtaaaactgc									
											
Pb293	KP780888	F: gcaaaagtggaccattcca	(CA)_20_	50	159-171	8	5.342	0.920	0.813	0.4681	43
		R:tcaccacactccgcaacat									
											
Pb318	KP780889	F: aaacccgaactgtccgtgtg	(TTTC)_24_	53	230-278	10	6.510	0.880	0.846	0.1927	0
		R: ggcttccgattgcacaacgaa									
											
Pb327	KP780890	F: cagcacagcgctcaccttcg	(GGACACA)_12_	56	259-345	16	10.684	0.920	0.906	0.1410	43
		R: tgattccgcagggcgtatgacg									
											
Pb344	KT699112	F: gcacagaaaccagaagagaca	(TAGA)_15_TTGA(TAGA)_21_	56	300-436	19	9.615	0.960	0.896	0.1447	0
		R: cgtctttggtggctatcagg									
											
Pb360	KT699113	F: gcagagacaattcagcctgg	(GT)_21_	56	190-204	7	5.682	0.760	0.824	0.3258	29
		R: gcagaagaagagtgcgtcat									

N, number of individual DNA samples included; TD, touchdown PCR; N_A_, number of alleles; H_o_, observed heterozygosity; H_e_, expected heterozygosity; P_HWE_, probability of deviation from Hardy-Weinberg equilibrium test; Success in cross-species amplification, number of samples that success in cross-species amplification out of 43 *P. megacephalus* samples.

**Table 2.** Characteristics of five polymorphic microsatellite loci in *P. megacephalus* (N = 27).

Locus Name	Allele Size range (bp)	N_A_	N_e_	Ho	He
Pb168	109-135	4	1.587	0.185	0.370
Pb214	176-204	11	6.568	0.741	0.848
Pb284	241-321	12	2.963	0.519	0.636
Pb293	136-146	4	1.776	0.407	0.437
Pb327	201-319	9	3.973	0.667	0.748

N, number of individual DNA samples included; NA, number of alleles; Ho, observed heterozygosity; He, expected heterozygosity.
